# Extract from the Sanitary Report to the Surgeon-General

**Published:** 1861

**Authors:** C. S. Tripler

**Affiliations:** U. S. A.


### EXTRACT FROM THE SANITARY REPORT TO THE SURGEON GENERAL.

By Surgeon C. S. TRIPLER, M. D., U. S. A.

For the quarter ending Dec. 31, 1860.

Among the recruits (four hundred in number at present, several large detachments having been sent off,) we have had more cases of venereal disease than usual—eleven of gonorrhœa and eight of chancre—most of them aggravated cases. All these men have been returned to duty or are convalescent.

The gonorrhœa has yielded to strict confinement to bed; saline purgatives in the first instance; the local use of cold water; injections of cerate of zinc, subnirate of bismuth, perchloride and persulphate of iron. In a few cases the balsam of copavia has been used. I am very much disposed to think the more I see of these cases, the principles of treatment do not differ from those of any other inflammation of a mucous membrane, though they may be admitted to be due to a specific cause; that rest throughout, local depletion, sedation and evaporants in the early stages, and such local applications as are known to restrain undue secretions from the mucous membranes in the subsequent stages, will be found all-sufficient in the great majority of cases. Sometimes, I admit, I have been compelled to resort to a mercurial alterative, from the obstinacy of the cases. It is possible a chancre may have existed in the uthera in these instances—I do not know the fact; I do not know, however, that no secondary symptoms have occurred in any of these cases while they have remained under my observation. The mercurial I employ for this form, is the bichloride, in doses of 1-16 to 1-8 grain three times a day. I have been pleased with the local use of the subnitrate of bismuth in a number of cases in the course of the last year. I think, however, that the preparations of iron I have indicated have been generally more satisfactory in their effects.—An alternation of these agents at the proper stage, but always combined with rest, I am persuaded, will seldom fail in effecting a permanent cure in from seven to fourteen days.

The cases of primary syphilis have been generally quite obstinate, but neither presenting unusual symptoms, nor calling for unusual medication. In three cases I have been obliged to perform circumcision for phymosis in order to expose concealed chancres. I may remark upon this operation, that, in performing it, I prefer to pass a director under the prepuce upon the dorsal median line and while an assistant draws the skin well back, to penetrate the integument with a sharp pointed bistoury and incise it. If the mucous membrane still protrudes when the skin is relaxed, I pass the director under it a second time and incise with the bistoury as before to the apex of the re-entering angle of the divided skin. Then with the scissors cut off the prepuce symmetrically on either side of the frenum to the extent desired. In this way, I think, we have less trouble with the mucous membrane than in the methods of either Ricord or Vidal.

During a part of the months of November and December we had a limited invasion of epidemic catarrh. It began on the 18th of Nov. and ceased 16th Dec.—just four weeks.—These patients were seized with chills, sometimes severe and protracted; engorgement of the mucous lining of the frontal sinuses, nares, pharynx, larynx, trachea and sometimes the bronchial tubes—fever for a few days and a rather obstinate cough. The treatment consisted of saline cathartics, antimonials, extract of wild cherry, or cyanide of potasium in combination with tincture of tolu and syrup of acacia, and demulcent drinks. The affection yields very readily.

We have had eleven cases of pneumonia thus far, this season—all of them severe, but fortunately none fatal. One of them was complicated with articular rheumatism.

These cases include what might be distinguished into capillary bronchitis, pleuro-pneumonia, bilious pneumonia, etc.—The existence of the disease was verified in every instance, both by the physical and rational signs. Sixteen days was the shortest period any of them remained upon the sick report; thirty to forty days have been usually required to re-establish strength sufficient to enable the men to return to duty without incurring too much risk of a relapse. I have treated, since I have been in charge of this hospital, nearly forty cases of this disease and have lost none. I have been informed by several of my friends that it has prevailed a great deal in this section of the country and has been very fatal. In referring to the records of this hospital, I find one hundred and twenty cases registered in five years, beginning in the autumn of 1847. But among these I find thirty-three cases which were returned to duty in from one to three days, and two more which were discharged in four days. These cases could scarcely have been pneumonia. Deducting them we have eighty-five still left—a very large number. Among these there are five deaths recorded, which gives a mortality of one in seventeen—only about one-third the mortality in civil hospitals. I do not know the exact system of treatment adopted in those cases. Blood-letting was sometimes used; cups occasionally but rarely; antimony the same.

Among my cases venesection was resorted to but once—the first case—and that without my authority. It was practised by my hospital sergeant, a very discreet and intelligent man, in the night, when the man was brought in, because he thought it a case of emergency, and he had seen it practised in such cases by my predecessor.

The mortality under my treatment has been zero; it cannot, therefore, be compared with any other table. I suppose a death must occur in the course of time, and then we can get a ratio less than infinity. As it stands, this mortality is less than any other reported. The fact is interesting in relation to the blood-letting controversy that has agitated the medical world since Prof. Bennett's novel views have been published. Without assenting to those views in mass, I admit that my experience in pneumonia at this point, one of its favorite localities, goes to confirm Professor Bennett's opinion as to the fact that general bleeding is unnecessary in this disease.—But, it will show at the same time, that neither is stimulation necessary.

My plan of treatment (I confine myself now to the thoracic difficulty—if other conditions exist, such as constipation, etc., they demand their own remedies) has been to confine the patient to bed until he is decidedly convalescent. I have seen more than one fatal relapse in the course of my life provoked by the patient's rising from bed when the disease was checked but not cured. Then wet cups at the seat of the disease as indicated by percussion and auscultation; and these repeated, following up the local lesion as it extends in the same lung or as it is propagated to the other. I have cupped a patient in this way two or three times a day for three or four days in succession. Dry cups have been frequently used during and after the same time. For constitutional treatment I have, until this winter, relied almost exclusively upon the tartrate of antimony—$\frac{1}{8}$ to $\frac{1}{2}$ a grain, as tolerance is established, every two hours, one hour, half-hour, as it can be borne. This winter I have used freely Norwood's tr. verat. virid. and with great satisfaction. Three cases have been treated with this alone, six with tart- antim. et pot., one with both, and one with veratrum followed by calomel and dover's powder, and a blister in its latter stages. The calomel and dover's powder were given because, notwithstanding the complete control of the heart's action manifested by the veratrum and the progressive resolution of the local engorgement as shown by auscultatien, constitutional irritation, insomnia and delirium supervened, persisted, and assumed a threatening aspeet. In this emergency I resorted to Stokes' plan, and substituted calomel and opium and a blister for the sedatives and local depletion employed in the earlier stages of the disease.

I have been careful always to endeavor to make the patient swallow a tablespoonful of strong animal broth frequently in the course of the day during the treatment. While the man is very ill he will resist this, but I feel persuaded I have seen very beneficial results from this course. Warm mucilaginous drinks are kept constantly at the bedside of the patient.

The cases which I have treated this year have been, eight upon the right side, one upon the left, and two double. They have all commenced at the inferior and lateral or posterier part of the lobe.—*Mar. and Virg. Journal.*

